# Longevity in the South Carolina Alzheimer’s disease registry

**DOI:** 10.3389/fneur.2024.1425495

**Published:** 2024-08-21

**Authors:** Maggi C. Miller, Eric Mishio Bawa, John R. Absher, Leonard Bonilha, Lesley A. Ross, Hye Won Chai, Nicholas J. Milano, Robert J. Adams

**Affiliations:** ^1^Department of Epidemiology and Biostatistics, Office for the Study of Aging, University of South Carolina, Columbia, SC, United States; ^2^Brain Health Network, University of South Carolina, Columbia, SC, United States; ^3^Department of Medicine, Division of Neurology, School of Medicine Greenville, University of South Carolina, Greenville, SC, United States; ^4^School of Health Research, Clemson University, Clemson, SC, United States; ^5^Department of Pharmacology, Physiology, Neuroscience, School of Medicine Columbia, University of South Carolina, Columbia, SC, United States; ^6^Department of Neurology, Medical University of South Carolina, Charleston, SC, United States

**Keywords:** Alzheimer’s disease, dementia, longevity, survival, registry

## Abstract

**Background:**

South Carolina has arguably the most robust Alzheimer’s Registry in the United States. For enhanced planning in both clinical practice and research and better utilization of the Registry data, it is important to understand survival after Registry entry. To this end, we conducted exploratory analyses to examine the patterns of longevity/survival in the South Carolina Alzheimer’s Disease Registry.

**Methods:**

The sample included 42,028 individuals in the South Carolina Alzheimer’s Disease Registry (SCADR). Participants were grouped into four cohorts based on their year of diagnosis. Longevity in the Registry (LIR), or the length of survival in the registry, was calculated based on the years of reported diagnosis and death.

**Results:**

The median LIR varied between 24 to 36 months depending on the cohort, with 75% of individuals in the three recent cohorts surviving for at least 12 months. Across all cohorts, 25% of the participants survived at least 60 months. The median LIR of females was longer than that of males. Individuals whose race was classified as Asian, American Indian, and other than listed had longer LIR compared to White, African American, and Hispanic individuals. Median LIR was shorter for Registry cases diagnosed at an earlier age (less than 65 years).

**Conclusion:**

Our data indicate that significant longevity is to be expected in the SCADR but that there is interesting variability which needs to be explored in subsequent studies. The SCADR is a rich data source prime for use in research studies and analyses.

## Introduction

Compared to the registries that track cancer, population-based registries engaged in the surveillance of Alzheimer’s Disease (AD) are much less common. Among the four AD registries that are established in the US, the South Carolina Alzheimer’s Disease Registry (SCADR), hereon referred to as SCADR or the Registry, is arguably the oldest and most comprehensive registry, beginning in 1988 (1). Cancer registries have set standards (2–4) and have proven to be a vital source of data that delineate the risk and preventive factors of the disease, the timing of diagnosis to treatment, access to care, and racial disparities in disease prevalence based on geographical location and religious affiliation (5). The SCADR identifies ADRD diagnosis information when the individual or their family members seek provider services. The SCADR does not capture clinical indicators such as markers for severity of the disease but it does obtain dates related to diagnosis and death. Work is still needed for AD registries in estimating survival, or Longevity in the Registry (LIR; how long an individual stays in the registry until death after entering into the registry with dementia diagnosis), as it can provide important information for enhanced care planning and informed decision-making in clinical practice (6). Additionally, a better understanding of differences in survival rates by demographic factors such as race, sex, and age is also pivotal in planning research studies including possible clinical trials in identifying vulnerable populations that are in need of more resources and support.

Interest in longevity is important for at least two reasons. First, assuming that being in the Registry does not directly impact survival, how long people live with more established diseases such as Alzheimer’s disease is an indicator of the efficiency of the medical system that cares for individuals with AD and physicians making the diagnosis of AD. In other words, longer longevity would imply that individuals with AD are either being diagnosed earlier or that interventions and treatments are effective in helping individuals live longer.

Secondly, the Registry maintains strict confidentiality requirements with existing data sources, however, the Registry staff have contact information and are allowed to contact families and physicians of persons reported to the Registry for additional data which is deidentified prior to being made available to public and private partners (7). As such, there are multiple data protection and approval processes needed to maintain patient confidentiality prior to gaining access to the Registry data. Whether a researcher would be inclined to go through these procedures may depend on the type of the research question and if Registry cases have some observable longevity in the Registry (as opposed to post-mortem identification). For researchers interested in using the Registry for the purposes of research or interventions, observable lived time or LIR for registry cases can be important in determining the “return on time investment.”

Therefore, the goal of the current study is to assess LIR in the SCADR and to examine differences in LIR across four different cohorts by demographic characteristics such as age, sex, and race/ethnicity. A better understanding of these factors will inform researchers in designing studies that utilize the SCADR as well as compare cohort effects of LIR.

## Methods

### Data

The data came from SCADR, a statewide registry of South Carolina residents diagnosed with or treated for ADRD. The Registry incorporates records from multiple sources that provide administrative data on inpatient hospitalizations, mental health records, Medicaid claims, emergency department visits, memory clinic encounters, vital records, and long-term care evaluations (1). Inclusion criteria for the study sample included an International Classification of Diseases, 10th revision, Clinical Modification (ICD-10-CM) coded medical record indicating AD, vascular dementia, mixed dementia, and other dementia diagnoses. Other dementia diagnoses include 14 other dementia-related diagnoses, including Parkinson’s disease, dementia with Lewy Bodies, frontotemporal and dementias related to traumatic brain injury, AIDS or alcohol or drug abuse (1).

### Study sample

**Figure 1:**
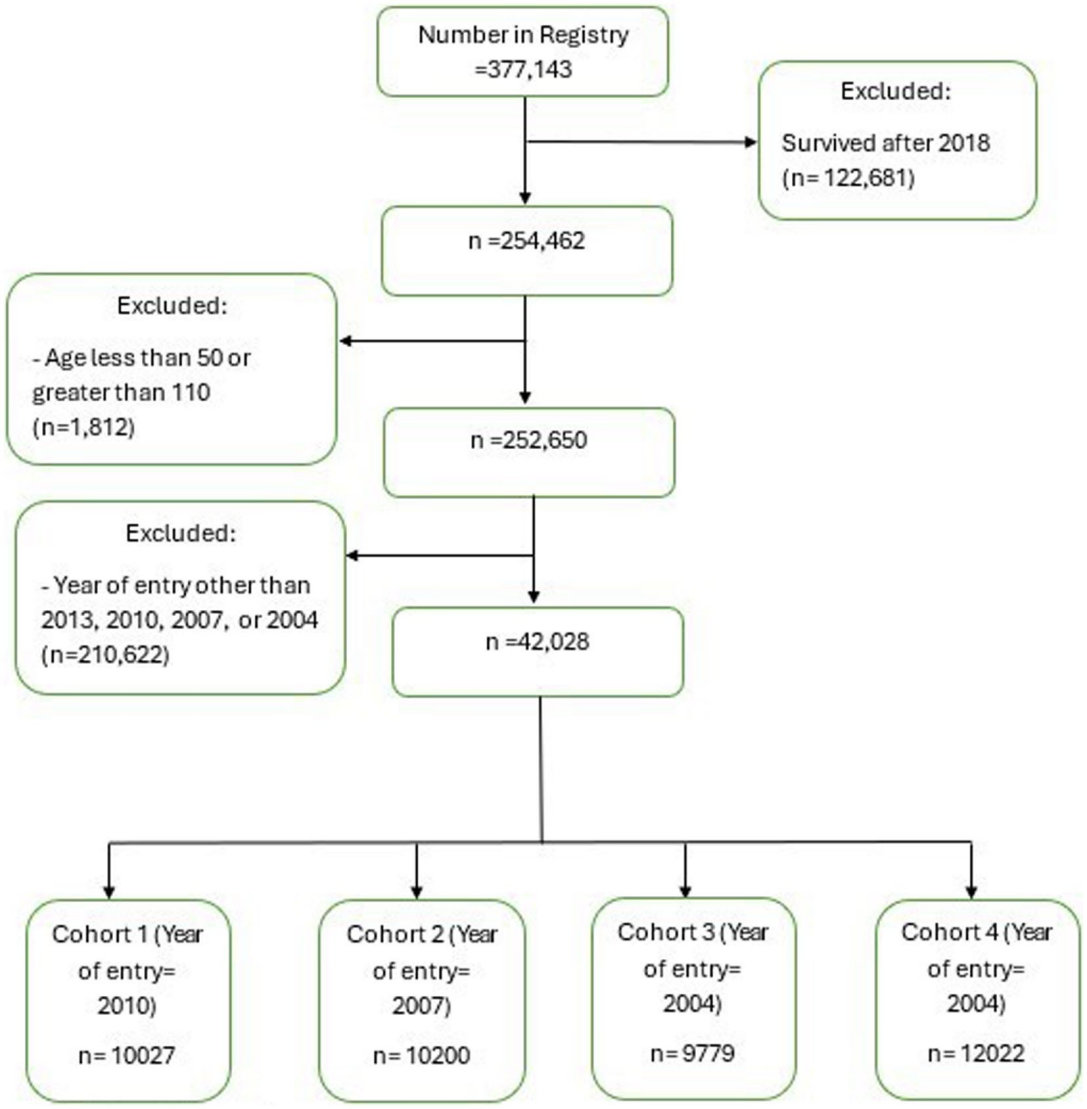
Participants included in the study.

The sample selection process followed the steps shown in Figure 1. The study sample initially consisted of individuals identified in SCADR (*n* = 377,143). This study then excluded participants who were alive until after 2018 (*n* = 122,681) and individuals younger than age 50 or older than age 110 (*n* = 1,812). Of the remaining 252,650 individuals, we further excluded a total of 210,622 individuals who were not a part of the four cohorts (2001, 2004, 2007, 2010) selected for this study (i.e., individuals who entered into the SCADR in years other than 2001, 2004, 2007, and 2010). This resulted in a final study sample of 42,028 individuals. The final sample was then divided into cohorts based on the year they entered the Registry. Individuals who entered the Registry in 2010 (*n* = 10,027), 2007 (*n* = 10,200), 2004 (*n* = 9,779), and 2001 (12,022) were classified as cohort 1, cohort 2, cohort 3 and cohort 4, respectively. If we had included cases who lived beyond 2018 (*n* = 122,681), only 11,167 (9.10%) would have been part of the final sample.

Since the average life expectancy after AD diagnosis ranges from 4 to 8 years according to the Alzheimer Disease Association (8), and was assumed to be stable across the study period, we investigated enrollment from 8 years prior to and including 2018, which is the most recent Registry data before the COVID-19 pandemic. We decided to select these four cohorts (2001, 2004, 2007, and 2010) to examine a general trend in LIR over time.

### Longevity in the registry

The outcome of interest for this study was the length of survival in months enrolled in the Registry, defined as Longevity in the Registry (LIR). LIR was examined by defining four groups of cohorts based on when the cases enrolled into the Registry. The year of entry into the Registry and the year of death were used to estimate LIR. In other words, LIR in months was calculated by subtracting the year of entry into the Registry from the year of death reported. The exact date of death was not available in the Registry due to confidentiality, so LIR was computed based on years and converted into months.

### Demographic characteristic

Indicators of demographic characteristics included in this study are the age of entry into the Registry in years, age group in years (<65, 65–74, 75–84, ≥85), sex (male or female), race (White, African American, Hispanic, Other) and dementia type (Alzheimer’s, vascular, mixed, other).

### Data analysis

Sample characteristics are summarized for each cohort using means and standard deviations for continuous variables, and frequencies and proportions for categorical variables. The One-Way ANOVA test was used to compare means across the four cohorts and the Chi-square test was used to compare the difference in proportions for categorical variables across cohorts. The LIR is presented using median and interquartile ranges for all four cohorts due to the skewed distribution of LIR. The median LIR was also calculated by sample characteristics. Due to the skewness of the data, the Kruskal-Wallis test was used to compare the LIR across cohorts. All data were deidentified and all analyses were carried out using SAS 9.4.

## Results

The sample characteristics are shown in Table 1. In all four cohorts, the mean age participants entered the Registry, meaning the age they were diagnosed with or treated for Alzheimer’s disease and related dementias (ADRD), was approximately 80 years. Generally, more than 75% of the participants in each cohort were 75 years or older with about 30% aged between 75 and 84 and about 45% being older than 85. The proportion of participants who were younger than 65 at the time of entry into the registry ranged from 3.7% in the 2001 cohort to 5.5% in the 2004 cohort. The majority in each cohort were females with proportions ranging from 62.4% in the 2010 cohort to 66.1% in the 2001 cohort. In terms of race, the majority were White individuals across all four cohorts, ranging from 66.38% in 2004 cohort to 70.52% in 2001 cohort. The most common dementia type for all four cohorts was AD. The proportion of participants with AD ranged from 62.59% in 2001 cohort to 68.43% in 2004 cohort.

**Table 1:** Characteristics of the participants in the study.

Variable	2010 cohort	2007 cohort	2004 cohort	2001 cohort	ANOVA/Chi-square
	*n* (%)	*n* (%)	*n* (%)	*n* (%)	*p*-value
*n*	10,027	10,200	9,779	12,022	
**Age at entry, years (mean, SD)**	79.52 ± 9.95	79.77 ± 9.73	79.05 ± 10.02	80.37 ± 9.24	<0.0001
**Age group, years**					<0.0001
< 65	545 (5.44)	506 (4.96)	539 (5.51)	444 (3.69)	
65–74	1,445 (14.41)	1,388 (13.61)	1,386 (14.17)	1,400 (11.65)	
75–84	3,106 (30.98)	3,392 (33.25)	3,394 (34.71)	4,480 (37.27)	
≥85	4,931 (49.18)	4,914 (48.18)	4,460 (45.61)	5,698 (47.40)	
Sex*					<0.0001
Male	3,653 (37.64)	3,768 (37.54)	3,499 (36.17)	4,047 (33.87)	
Female	6,052 (62.36)	6,269 (62.46)	6,175 (63.83)	7,901 (66.13)	
**Race**					<0.0001
White	6,699 (66.81)	6,990 (68.53)	6,491 (66.38)	8,478 (70.52)	
African American	2,319 (23.13)	2,630 (25.78)	2,835 (28.99)	3,335 (27.74)	
Hispanic	33 (0.33)	41 (0.40)	40 (0.41)	24 (0.20)	
Other	976 (9.73)	539 (5.28)	413 (4.22)	185 (1.54)	
**Dementia type**					<0.0001
Alzheimer’s	6,556 (65.38)	6,678 (65.45)	6,692 (68.43)	7,524 (62.59)	
Vascular	1,053 (10.50)	1,091 (10.70)	1,049 (10.73)	1,413 (11.75)	
Mixed	373 (3.72)	484 (4.75)	662 (6.77)	658 (5.47)	
Other	2,045 (20.39)	1,949 (19.11)	1,376 (14.07)	2,427 (20.19)	

*The sex variable has missing data, so the total is different from other variables reported here.

Table 2 presents median and interquartile LIRs by cohort and demographic characteristics. Overall median LIR in the Registry was 36 months for both the 2010 and 2004 cohorts, and 24 months for both the 2007 and 2001 cohorts. Lower quartile (25%) LIR for the three recent cohorts (2004, 2007, and 2010) was 12 months, which indicate that 75% of the Registry cases for these cohorts survived for 12 months or more. The minimum LIR for the upper 25% of participants was 72 months for those who entered the Registry in 2004, and 60 months for those who entered in 2010, 2007, and 2001.

**Table 2:** Median longevity in registry (LIR) by Cohort and demographic characteristics (in months).

Variable	2010 cohort	2007 cohort	2004 cohort	2001 cohort	Kruskal-Wallis
	(*n* = 10,027)	(*n* = 10,200)	(*n* = 9,779)	(*n* = 12,022)	
	Median (IQR)	Median (IQR)	Median (IQR)	Median (IQR)	*p*-value
**Overall LIR**	36 (12, 60)	24 (12, 60)	36 (12, 72)	24 (0, 60)	**<0.0001**
**Age group, years**					
< 65	24 (12, 48)	12 (0, 36)	12 (0, 48)	12 (0, 48)	**<0.0001**
65–74	36 (12, 72)	24 (0, 60)	24 (12, 72)	12 (0, 48)	**<0.0001**
75–84	36 (12, 60)	24 (12, 60)	24 (12, 60)	12 (0, 48)	**<0.0001**
≥85	36 (24, 72)	36 (12, 72)	36 (12, 72)	24 (12, 60)	**<0.0001**
**Sex**					
Male	36 (12, 60)	24 (12, 60)	24 (0, 60)	12 (0, 48)	**<0.0001**
Female	36 (12, 60)	36 (12, 60)	36 (12, 72)	24 (0, 60)	**<0.0001**
**Race**					
White	36 (12, 60)	24 (12, 60)	24 (12, 60)	24 (0, 48)	**<0.0001**
African American	36 (12, 72)	36 (12, 72)	36 (12, 84)	24 (0, 60)	**<0.0001**
Hispanic	36 (24, 72)	24 (0, 60)	12 (0, 54)	24 (12, 48)	0.0721
Other	48 (24, 96)	60 (12, 120)	60 (24, 120)	84 (36, 156)	**<0.0001**
**Dementia type**					
Alzheimer’s	36 (24, 72)	24 (12, 72)	24 (12, 72)	24 (12, 60)	**<0.0001**
Vascular	36 (12, 60)	24 (12, 60)	24 (12, 72)	12 (0, 48)	**<0.0001**
Mixed	36 (12, 60)	24 (12, 60)	24 (12, 48)	36 (12, 60)	**<0.0001**
Other	36 (24, 60)	24 (12, 60)	36 (12, 84)	12 (0, 36)	**<0.0001**

IQR, interquartile range represented with the lower and upper quartiles (25 and 75%).

When stratified by age, overall patterns showed that median LIR for registry cases who were younger than 65 at the time of entry into the Registry was half the median LIR of those who entered the Registry after the age of 65. For example, in 2004 and 2007 cohorts, median LIR for those who entered into the Registry before the age of 65 was 12 months, while the median LIR for those who entered between the ages 65 and 84 was 24 months. Generally, among the cases who entered the Registry before the age of 65, 50% survived beyond 12 months and 25% died in the year they entered the Registry. In the 2010, 2007 and 2004 cohorts, 50% of cases who were aged 85 years or older at the time of the entry into the Registry survived beyond 36 months. In these same cohorts, 25% of cases aged 85 years or older at the time of the entry survived beyond 72 months.

In terms of sex, females survived longer in the Registry compared to males. In 2007, 2004, and 2001 cohorts, the median LIR for females was longer than the LIR for males by 12 months. For example, while median LIRs for females in the 2007 and 2004 cohorts were 36 months, median LIRs for males in these cohorts were 24 months. Similarly, in the 2001 cohort, median LIR for females was 24 months while the median LIR for males was 12 months.

As for race, registry cases whose race was classified as ‘other’ (including Asian, American Indian, and other than listed) had longer LIR than White, African American, and Hispanic cases. Their median LIR ranged from 48 months to 84 months, while the median LIRs for the other three racial groups ranged from 24 months to 36 months. No apparent patterns emerged when stratified by dementia type, other than that the median LIRs tended to be the same across all types of dementia in the three recent cohorts.

## Discussion

Given the importance of LIR of AD cases in planning clinical practice and research, this study aimed to examine the LIR of four cohorts from SCADR by demographic characteristics. This study found that the median LIR in SCADR ranged from 24 to 36 months. Our estimated median LIR falls within an estimated survival range of 1.3 to 7.2 years (15.6–86.4 months) reported in an earlier systematic review (9). It is also within the median survival range of 1.9 to 6.7 years (22.8–80.4 months) reported by a cohort study that estimated the survival after a diagnosis of dementia in primary care (10). Survival after dementia diagnosis varies widely and depends considerably on numerous factors (9). However, the findings of this study do contradict other studies that report longer longevity. A study conducted with electronic health records in the Netherlands examined the trajectory of individuals with dementia after diagnosis. This study reported a median survival of 5 years (60 months) (11). Our findings may differ from the study in the Netherlands due to the lower life expectancy in the USA compared to the Netherlands and other developed countries (12). Another study that estimated the survival following a diagnosis of AD reported a median survival range of 3.4 to 8.3 years (40.8–99.6 months) (13). This study may have shown longer survival due to a different study design. They recruited dementia-free participants, identified incident cases, and followed them to estimate survival. A systematic review on survival in dementia also reported a median survival time range of 3.3 to 11.7 years (39.6 months to 140.4 months) (14). The difference in median survival between our study and this review could be due to the design, data sources and sample size of the studies considered in the review. Most of the studies in this review were cohort studies where disease-free individuals were followed, none of the studies in this review used data from a registry, and the study with the largest sample size in this review had only 2,923 participants. The current study relied on data from the Registry where the majority of cases are likely to be identified at the later stages of the disease. Individuals with ADRD enter the Registry when they or their family members seek provider services. Due to the denial and stigma related to dementia, many individuals and families wait until a crisis occurs to seek treatment which leads to late diagnosis. Thus, if Registry cases are identified earlier in the disease course, they would be expected to survive longer which would allow for the potential to test disease modifying treatments to prolong longevity/survival.

Our findings related to gender were consistent with other studies. Females had a longer longevity in the Registry compared to males. Other studies also report longer median survival for females (6, 11). This may be due to the longer life expectancy of females compared to males, which is often explained by the higher likelihood of females engaging in health-promoting behaviors compared to males (15). When compared to men, women generally engage more in healthy-seeking behaviour and less in risky behaviours. For instance, women eat healthier, drink less alcohol, smoke less, visit doctors more and record lower suicide rates (16–19). Indeed, studies consistently find that females, compared to males, engage less in risky health behaviors such as drinking and smoking (20), are more willing to have screening health checks, seek support and information from medical practitioners, and adhere to medical advice (21). These health behaviors together may be protective against the progression of ADRD symptoms and allow females to have longer LIR compared to males. In order to better understand the sex differences in LIR, future studies need to investigate the biological, psychological, and social mechanisms that could lead to such differences in longevity by sex.

In the current study, Registry cases whose race was classified as “other” survived longer compared to White, African American, and Hispanic cases. The “other” race category comprises of Asian, American Indian, and other than listed. These findings are consistent with work conducted in Northern California examining survival in five racial groups after a dementia diagnosis (22). This study found that survival after dementia for Asian Americans was longer than that of White individuals, African Americans, and Latinos (22). Similarly, a study on Medicare beneficiaries also reported that Asians/Pacific Islanders have longer post-dementia survival than that of White individuals and African Americans (23). These findings could be due to the mortality crossover in later life, where other races have a mortality advantage over White individuals (24, 25). While our findings on race were consistent with the findings of other studies on Asian Americans, our findings contradicted the findings of other studies on Indians/Alaska natives. Two studies reported that Indians/Alaska natives had a longer survival time than White individuals but no other races (22, 23) The difference may be due to the categorization of race. The Registry combines American Indians and Asian Americans into one group, while other studies separated them. Further investigation into the social determinants of health could be informative in better understanding the racial patterns of LIR. For example, it is possible that longer LIR among Asian Americans is in part attributable to a relatively higher education level of Asians compared to other racial groups (26), where education may function as a resource that delays further cognitive and functional decline among individuals with ADRD. It is also possible that the longer LIR among the “other” race could be the benefit of social support. For instance, Asian Americans could benefit from cultural beliefs such as valuing social ties with family and friends (27, 28). Social connections and support are strong determinants of health and well-being as they buffer the effect of social isolation, lower the adverse effects of stress, and increase positive emotions (29).

The estimated median survival was lower for Registry cases diagnosed at an earlier age with ADRD. A retrospective study in Australia also found that individuals diagnosed with ADRD before the age of 65 with a history of diabetes died two times faster than those without diabetes (30). However, the effect of diabetes on mortality was absent among participants diagnosed with ADRD at 85 years or older (30). This suggests that comorbidities among younger individuals with ADRD could accelerate mortality, calling for interventions to attenuate the effect of comorbidities. Among the Registry cases in this study, individuals aged younger than 64 years and those between the ages 65 and 74 accounted for more than 70% of alcohol-related dementia cases in all four cohorts. In addition, the majority of participants with HIV in this study were younger than 65 (see Supplementary Table S1). Our finding on median survival for cases diagnosed at an earlier age is different from previous findings on survival after dementia diagnosis. A cohort study in the UK using data from the Health Improvement Network found that median survival decreased with age of dementia diagnosis (10). The Baltimore Longitudinal Study of Aging also reported a longer survival for participants diagnosed with Alzheimer’s disease at age 65 compared to those diagnosed at older ages (13). Our contradictory findings may be due to the distribution *of* the types of dementia in the SCADR as well as the presence of comorbidities.

When comparing LIRs across the four cohorts, our results showed that LIRs for later cohorts tended to be longer compared to those of earlier cohorts. For example, while the overall lower 25% and median LIRs for the 2001 cohort were 0 and 24 months respectively, the LIRs for the 2010 cohort were 12 and 36 months. These patterns remained the same in general when divided by age and gender. One possible explanation could be that compared to the earlier cohorts, later cohorts may have easier access to information or care that allows for an early detection and better management of dementia, which may be associated with longer survival in the registry for later cohorts.

Our results show that the mean age of entry into the Registry was approximately 80 years. This is consistent with the findings of a study conducted in Sweden that predicted survival after an Alzheimer’s diagnosis using a national dementia registry. The median age at diagnosis was 82.9 years and 79.6 years for those in primary care and memory clinic respectively (6). An older age of diagnosis or entry into the registry is indicative of recognizing or diagnosing cases in the middle or later stages of the disease. Most dementia diagnoses are made when the disease has progressed to moderate or severe stages (31). In a retrospective cohort study at the Spectrum Health Medical Group Neurocognitive Clinic, 78.9% of the reviewed cases had moderate to severe dementia at the time of diagnosis (32). Diagnosis at the mid- or late stage can be due to the delay between the onset of symptoms and diagnosis. This gap could be attributed to the absence of accurate and reliable ADRD biomarkers, the mistaken belief that cognitive problems are a part of normal ageing, and the denial or lack of recognition by clinicians, patients, and caregivers (31, 32). Early diagnosis of dementia has benefits such as thorough evaluation of a patient for reversible causes of memory loss and an improved quality of life of both the patient and the families (33). Diagnosis at a later stage leads to higher levels of disability while receiving care (8).

A major strength of this study is the use of a large sample from a population-based registry using four cohorts spanning up to 15 years of data. In addition, the population-based design of this study may make it generalizable to regions with demographically similar populations with ADRD. One significant limitation is that the LIR in this study is based on years of diagnosis and death, which limits the precise calculation of survival in months or days. In addition, while statistical significance is achieved in the comparison of LIR across the cohort, this significance may not be practical and could be due to the large sample size in each cohort. Also, because this study only selected four cohorts from the Registry, result may differ with inclusion of other cohorts. Lastly, because this study focused on providing descriptive analyses of survival after dementia diagnosis, future studies should conduct survival analyses using Cox proportional hazard models to have more in-depth understanding of the protective and risk factors of LIR.

In conclusion, our results show that the median longevity in the registry ranged from 24 to 36 months and 75% of ADRD cases survived more than 12 months in the registry. Even the age group with the shortest LIR, those diagnosed with dementia before the age of 65, still have a long enough follow-up time that could be useful for research studies with 50% of the sample surviving more than a year. Our results confirm that the SCADR is a valuable starting point for acquiring useful and necessary information for recruiting ADRD cases for participation in research studies, including clinical trials.

## Data Availability

The data analyzed in this study is subject to the following licenses/restrictions: there is an application process to obtain this data. Requests to access these datasets should be directed to https://osa-sc.org/programs/alzheimers-disease-registry.
