# Supplementary material for: Longevity in the South Carolina Alzheimer’s disease registry
Source: Front Neurol. 2024 Aug 21;15:1425495. doi: 10.3389/fneur.2024.1425495 (PMC11371588; doi:10.3389/fneur.2024.1425495)
Supplement: Supplementary file 1 [file Table_1.docx]

**Supplementary Table 1**

| Cohort by age group | Alcohol-related dementia | Creutzfeldt-Jakob Disease | HIV |
| --- | --- | --- | --- |
| 2010 cohort |  |  |  |
| <65 years | 84 (38.18) | 2 (100) | 9 (56.25) |
| 65 – 74 years | 86 (39.09) | 0 (0) | 4 (25) |
| 75 – 84 years | 39 (17.73) | 0 | 3 (18.75) |
| 85 years or older | 11 (5.0) | 0 | 0(0) |
| Total | 220 | 2 | 16 |
|  |  |  |  |
| 2007 cohort |  |  |  |
| <65 years | 66 (34.38) | 1 (100) | 7 (53.85) |
| 65 – 74 years | 69 (35.94) | 0 (0) | 2 (15.38) |
| 75 – 84 years | 47 (24.48) | 0 (0) | 3 (23.08) |
| 85 years or older | 10 (5.21) | 0 (0) | 1 (.69) |
| Total | 192 | 1 | 13 |
|  |  |  |  |
| 2004 cohort |  |  |  |
| <65 years | 52 (35.96) | 0 (0) | 11 (64.71) |
| 65 – 74 years | 51 (35.17) | 0 (0) | 5 (29.41) |
| 75 – 84 years | 36 (24.83) | 0 (0) | 0 (0) |
| 85 years or older | 6 (4.14) | 0 (0) | 1 (5.88) |
| Total | 145 | 0 | 17 |
|  |  |  |  |
| 2001 cohort |  |  |  |
| <65 years | 15 (27.78) | 0 (0) | 7 (53.85) |
| 65 – 74 years | 20 (37.04) | 0 (0) | 3 (23.08) |
| 75 – 84 years | 13 (24.07) | 1 (100) | 3 (23.08) |
| 85 years or older | 6 (11.11) | 0 (0) | 0 (0) |
| Total | 54 | 1 | 13 |
